# Nanoscopic surfactant behavior of the porin MspA in aqueous media

**DOI:** 10.3762/bjnano.4.30

**Published:** 2013-04-25

**Authors:** Ayomi S Perera, Hongwang Wang, Tej B Shrestha, Deryl L Troyer, Stefan H Bossmann

**Affiliations:** 1 Kansas State University, Department of Chemistry, CBC Building 201, Manhattan, KS 66506, USA; 2 Kansas State University, Department of Anatomy & Physiology, Coles 130, Manhattan, KS 66506, USA

**Keywords:** charge-interaction, hydrophobic interaction, liposome-type cluster, protein cluster, supramolecular, temperature influence, zeta potential

## Abstract

The mycobacterial porin MspA is one of the most stable channel proteins known to date. MspA forms vesicles at low concentrations in aqueous buffers. Evidence from dynamic light scattering, transmission electron microscopy and zeta-potential measurements by electrophoretic light scattering indicate that MspA behaves like a nanoscale surfactant. The extreme thermostability of MspA allows these investigations to be carried out at temperatures as high as 343 K, at which most other proteins would quickly denature. The principles of vesicle formation of MspA as a function of temperature and the underlying thermodynamic factors are discussed here. The results obtained provide crucial evidence in support of the hypothesis that, during vesicle formation, nanoscopic surfactant molecules, such as MspA, deviate from the principles underlined in classical surface chemistry.

## Introduction

**Figure 1:**
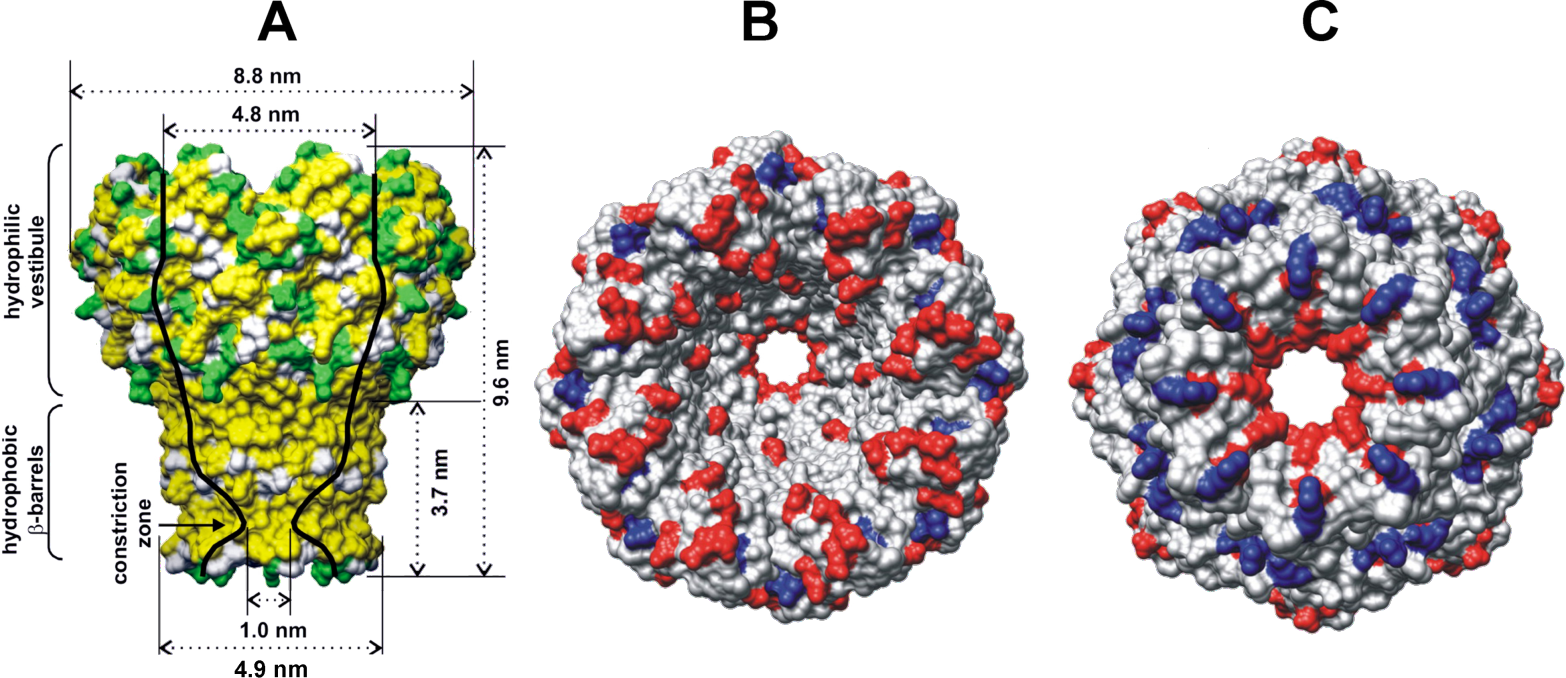
The structure of the homo-octameric mycobacterial porin MspA. (A) MspA is 9.6 nm in length and 8.8 nm in width. Its “docking zone”, which is formed by hydrophobic β-barrels, is located at the “stem”. Reproduced with permission from [2]. Copyright 2004 The American Association for the Advancement of Science. (B) Structural model of the MspA pore viewed from the top. Negatively and positively charged amino acids are shown in red and blue, respectively. Other amino acids are shown in gray. (C) MspA pore viewed from the bottom. (B) and (C) were adapted from [18] using the UCSF Chimera software. Chimera is developed by the Resource for Biocomputing, Visualization, and Informatics at the University of California, San Francisco (supported by NIGMS P41-GM103311) [19].

The homo-octameric porin MspA from *Mycobacterium smegmatis* is one of the most stable proteins known to date [1]. Due to its size and unique structure [2], its resistance to temperature and pH-changes, and its stability in nonaqueous solvents [3], MspA has become a versatile tool in bio-nanotechnology. MspA is able to reconstitute within phospholipid double layers [4] and polymer layers on surfaces [5]. Moreover, this protein can stand alone on surfaces without a supporting polymer or double layer [6]. It is capable of binding gold nanoparticles [6–7] and Ruthenium(II) polypyridyl complexes [8]. In fact, the binding of so-called “channel blockers” near the constriction zone of MspA has been discussed as a new strategy to fight mycobacterial infections, such as tuberculosis [8–9]. Although the presence of MspA homo-octamers on surfaces has been unambiguously proven by using transmission electron microscopy (TEM) [5], atomic force microscopy (AFM) [6], and electrochemical techniques [10], only very little is known about the three-dimensional clustering behavior of MspA in aqueous phase. Engelhardt et al. have established by using high-resolution TEM that MspA forms micelles and linear aggregates on surfaces showing a zipper-like pattern in the absence of surfactants, and that MspA is able to reconstitute in dimyristoyl phosphatidylcholine (DMPC) vesicles in the presence of a HEPES (pH 7.5)/NaN_3_ buffer [11]. The formation of this typical zipper-like pattern is achieved through the interaction of the strongly hydrophobic docking zones of MspA (Figure 1, see below), thus shielding the stems of the proteins from water.

This study is concerned with the 3D-aggregation behavior of MspA in aqueous buffers, further expanding the pioneering work of Engelhardt et al. In 1× PBS (phosphate-buffered saline), MspA is capable of forming vesicles in the absence of added surfactant. Owing to the great thermal stability of MspA [3], we were able to study the influence of ionic strength and especially the temperature on the size of the MspA-vesicles and their zeta potentials, ζ. The influence of temperature on the 3D-aggregation behavior of peptides is rarely discussed, because the temperature is well defined in many living organisms and only a few proteins do not denature at higher temperatures. α-Hemolysin from *Staphylococcus aureus* forms heptameric transmembrane pores that are stable over a wide pH range and at temperatures up to 60 °C [12]. However, heptameric α-hemolysin pores are not stable without a stabilizing membrane. Therefore, it can be expected that clusters of monomers (not heptamers) will be formed at higher temperatures in the absence of a membrane. Principally, the same behavior, albeit at lower temperatures (*T* > 40 °C), can be anticipated for the protective antigen part of the anthrax toxin from *Bacillus subtilis*/*Bacillus anthracis*, which forms heptameric and octameric oligomers [13]. In the near future, designer proteins with tailored biophysical properties will become increasingly available [14], and therefore, the influence of temperature on their supramolecular aggregation behavior will become more significant. Recently, the temperature dependence of the dynamics of several proteins has been studied by Förster resonance energy transfer (FRET) [15–17]. This study was intended to demonstrate the potential of using dynamic light scattering (DLS) and the measurement of zeta potentials when studying the supramolecular aggregation of proteins.

## Results and Discussion

MspA (porin A from *M. smegmatis*), an octameric channel protein (184 amino acids, *M*_w_ = 155,248 Da [20]) is isolated from the outer cell wall of *M. smegmatis*, a species of nonpathogenic mycobacteria commonly found in soil [21]. The structure of MspA has been studied extensively and bares no significant resemblance to any other protein known to date [2]. X-ray studies performed on a mutant MspA strain have provided a complex, detailed structural analysis [2]. Extraction of MspA is carried out using nonionic detergents and temperatures as high as 90 °C [22]. Remarkably, this porin retains its pore forming ability even after being exposed to harsh physical conditions such as temperatures up to 100 °C in SDS [23] and extreme pH values from 2 to 14 [1]. In fact, high temperature has been a crucial factor in determining the purity of MspA extracts, as other proteins were denatured and removed by these conditions. Consequently, MspA has been classified as the most stable channel-forming protein known so far. These findings make MspA especially suited for the study of the influence of temperature on supramolecular aggregation, as it is known to withstand drastic chemical conditions without denaturation.

**Figure 2:**
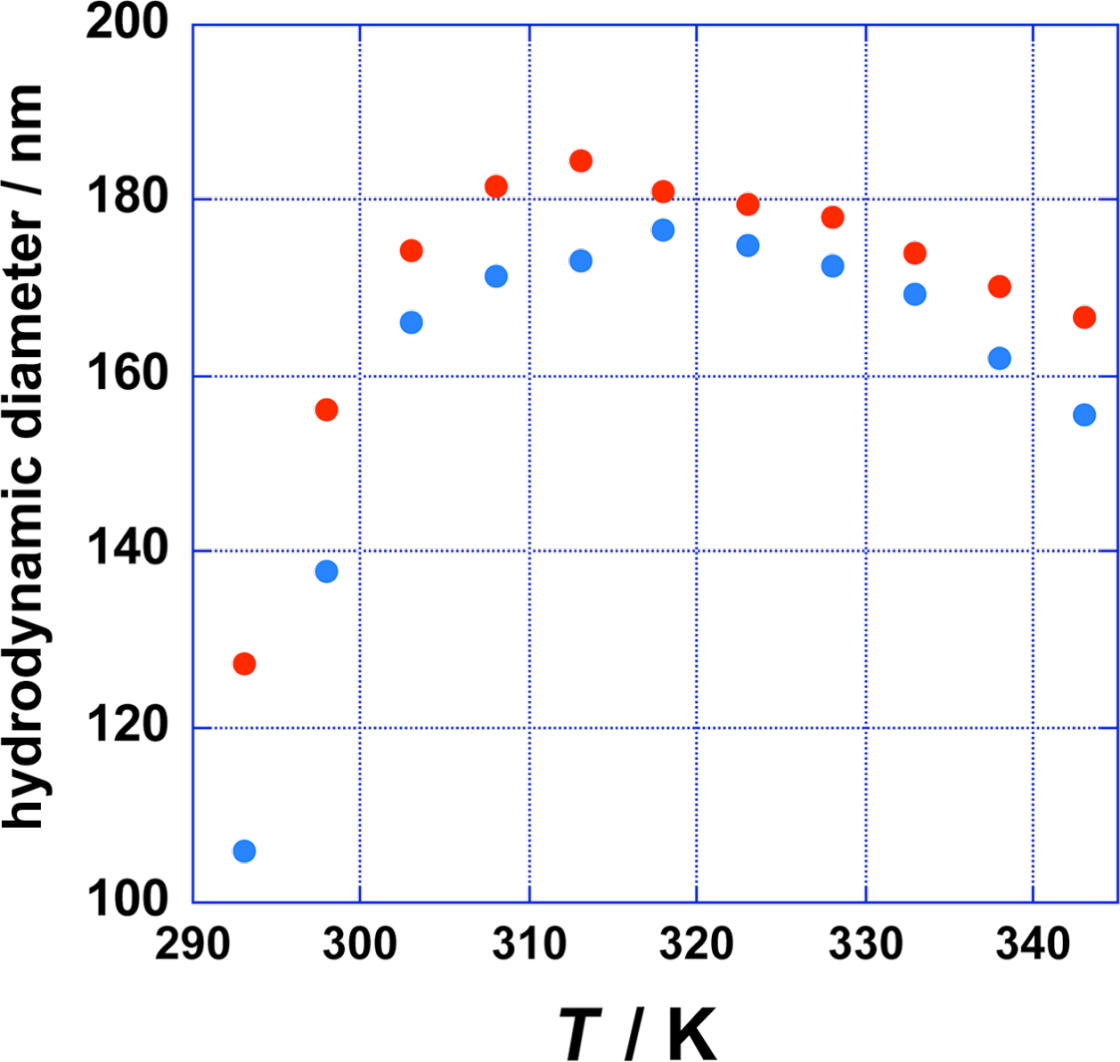
Hydrodynamic diameter of MspA aggregates as a function of temperature, measured by dynamic light scattering (DLS): blue: MspA (1.688 × 10^−5^ mg·mL^−1^) in (5 × 10^−5^)× PBS; red: MspA (1.688 × 10^−5^ mg·mL^−1^) in 1× PBS. The relative experimental error in diameter has been determined to be ±8 nm. Typical polydispersities of the formed supramolecular aggregates are provided in Supporting Information File 1. PBS consists of 8.0 g NaCl, 0.20 g KCl, 1.44 g Na_2_HPO_4_ and 0.24 g KH_2_PO_4_ in 1 L H_2_O, pH 7.40.

The MspA-octamer is formed by 160 negatively charged and 64 positively charged amino acids [2]. R165 and E63/E127, as well as R161 and E39, form salt bridges, which greatly stabilize its tertiary structure (R: arginine, E: glutamic acid) [2]. As a result, 136 negatively charged and 48 positively charged amino acids are accessible at the surface. Whereas the negative charges are predominantly found within the interior of the “goblet”, positive charges are concentrated in the stem and the periplasmatic loop region of MspA (Figure 1B and Figure 1C). We have investigated the aggregation of individual MspA in aqueous solutions ((5 × 10^−5^)× PBS and 1× PBS) as a function of temperature. The results are summarized in Figure 2. MspA shows a distinct tendency to aggregate independently of the ionic strength of the surrounding medium.

The maxima in hydrodynamic diameter of the supramolecular structures formed were observed at 312 K (standard PBS) and 318 K (diluted PBS). The diameters of these aggregates were in both cases very close to 180 nm and indistinguishable due to experimental error. Since the aggregation proceeds independently of the ionic strength of the medium, it is our paradigm that hydrophobic aggregation is the major mechanism behind the observed aggregation behavior of MspA. In applying a semiquantitative predictive model of forming supramolecular aggregates to MspA [24], we calculated the packing parameter

[1]
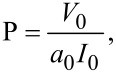


where *V*_0_ is the surfactant tail volume, *a*_0_ the area at the aggregate interface and *I*_0_ the tail length.

**Figure 3:**
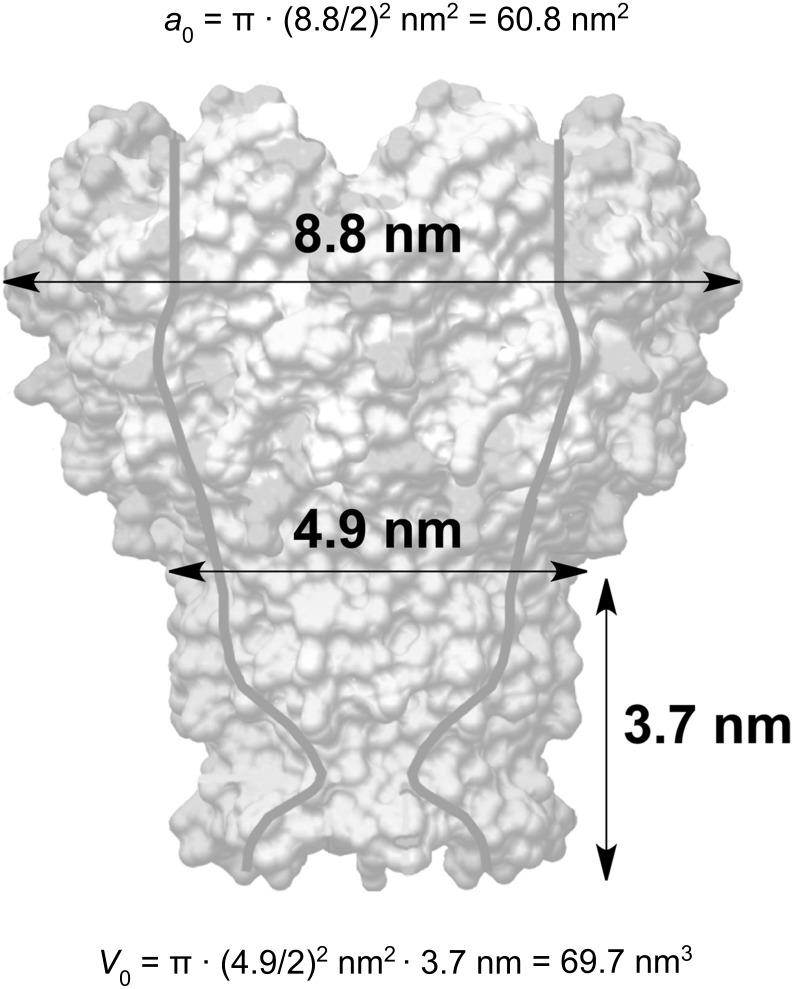
Geometric calculations based on the crystal structure of MspA [2].

Using the geometric parameters of MspA, we calculated *V*_0_ = 69.7 nm^3^ (the geometric dimensions of the “docking region” are 3.7 nm in length (*I*_0_) and 4.9 nm in diameter [2], see Figure 3), and *a*_0_ = 60.8 nm^2^.

**Figure 4:**
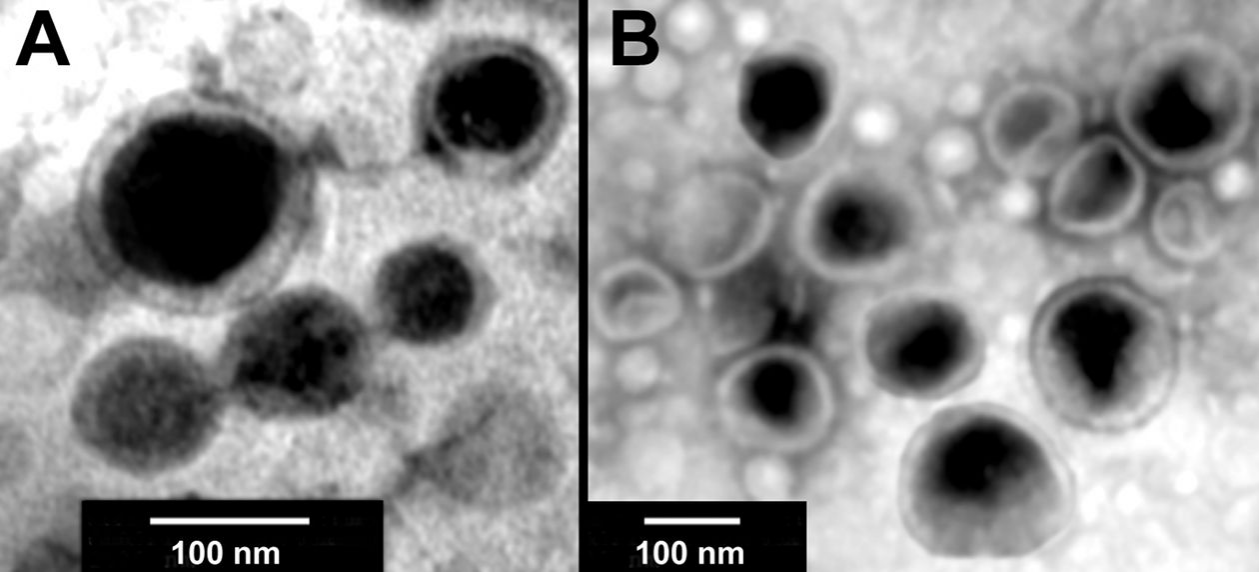
TEM of vesicles formed from MspA on a carbon-coated 200-mesh copper grid. A: MspA vesicles formed in (5 × 10^−5^)× PBS at 312 K (after deposition and in high vacuum on Cu). B: MspA vesicles formed in 1× PBS under analogous conditions.

The packing parameter of MspA is 0.31, which is indicative of surfactants forming spherical or ellipsoidal micelles. To our surprise, TEM characterization of MspA aggregates clearly indicated the formation of vesicles (Figure 4). However, vesicles are typically formed by surfactant bilayers featuring a packing parameter in the range of 0.5 to 1.0 [25].

This discrepancy requires a discussion. As discussed in the introduction, MspA forms linear aggregates in a zipper-like pattern on surfaces [11]. This behavior is indicative of a packing parameter that is very close to 1.0 [19]. Whereas the “docking zone” of MspA is formed by very stable hydrophobic β-barrels, the hydrophilic vestibule (the “head” of the surfactant) can potentially be deformed when single MspA proteins aggregate. Protein deformation is often observed during crystallization [26]. The formation of a bilayer is evidence for attractive interactions between MspA units. Predicting the geometry of supramolecular aggregates formed by one type of surfactant is to assume that the charged head groups show charge- and/or sterical repulsion [19]. However, the observed formation of vesicles indicates that the interactions of the vestibules are attractive. Furthermore, the formation of vesicles is not a function of ionic strength, as Figure 4 indicates, as MspA forms vesicles in both diluted and 1× PBS in a similar manner. This supports the mechanistic assumption that an efficient charge repulsion between the head groups of MspA is not observed.

### Aggregation number as a function of vesicle radius

We have calculated the aggregation number *N* of MspA-octamers that form a unilamellar vesicle as a function of the diameter of the vesicles according to Equation 2.

**Figure 5:**
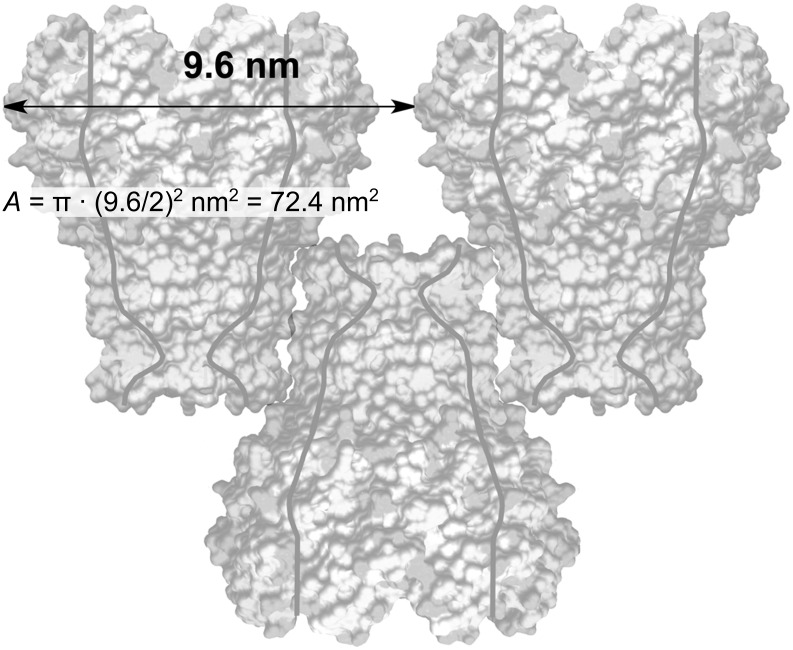
Distance between two neighboring MspA octamers in the outer layer of the vesicle’s double layer, and the effective size of MspA within that layer.

[2]
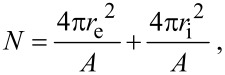


with *r*_e_ being the external radius of the vesicle (diameter divided by 2), *r*_i_ the inner radius of the vesicle (*r*_i_ = *r*_e_^2^ – 2·(*L*_MspA_ − *L*_dz_), *L*_MspA_: length of MspA = 9.6 nm; *L*_dz_: length of the docking zone = 3.7 nm, see Figure 1 and Figure 3), and *A* the area occupied by one MspA-octamer (*A* = 72.4 nm^2^). This calculation is based on the assumption that the docking zones are in contact in the vesicles' double layer. This interaction causes the centers of MspA within either the external or the internal layer to be 9.6 nm apart from each other, forming a simple packing pattern (Figure 5). The largest diameter of MspA is 8.8 nm [2].

**Figure 6:**
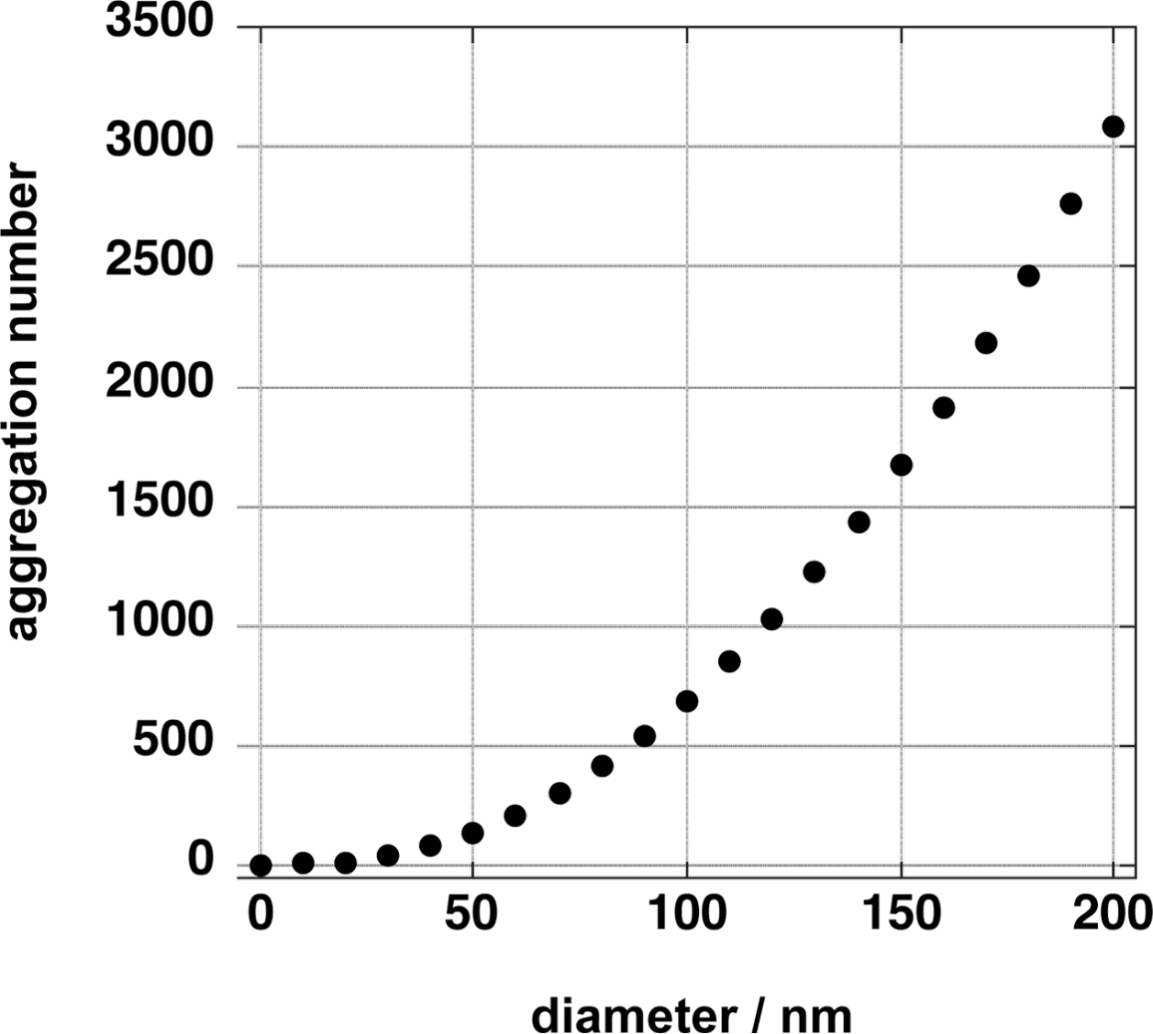
Estimated number of MspA-octamers forming a unilamellar vesicle (the presence of one MspA double layer is assumed) as a function of vesicle radius, according to Equation 2.

The inner radius *r*_i_ is smaller than the external radius *r*_e_ by twice the length of MspA minus the extension of the docking zone, because MspA forms aggregates showing a zipper-like pattern in which the hydrophobic docking zones are in contact with each other [11]. According to Equation 2 and Figure 6, the aggregation number *N* varies between *N* = 1395 (*d* = 138 nm) and *N* = 2470 (*d* = 180 nm) for the diameters reported in Figure 2.

### The hydrophobic effect is responsible for vesicle formation by MspA

We describe the self-assembly process by the free energy model originally developed by C. Tanford [27] and we assume that the residual contact of the water with the hydrophobic constriction zone is negligible after vesicle formation. Then the change in the chemical potential (Δμ°) during supramolecular aggregation is dependent on the transfer of MspA from the aqueous phase into the MspA-bilayer and the interaction of the head groups.

[3]
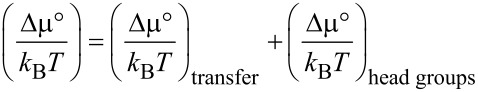


The term (Δµº/*k*_B_*T*)_transfer_ is negative, because the solvation of extended hydrophobic surfaces has a disruptive effect on the water structure. Whereas the hydrogen bond network of water around an alkane of modest length (e.g., C_6_H_14_) is not distorted significantly, the solvation of extended hydrophobic structures has a disruptive effect on the water structure because it prohibits the formation of an extended hydrogen bonding network. Huang and Chandler have established that the excess chemical potential decreases monotonically with temperature for structures with radii greater than 1 nm, as is the case with the “docking zone” of MspA (*r* = 1.85 nm) [28].

The term (Δµº/*k*_B_*T*)_head groups_ describes the energetic contribution arising from the interactions of the vestibules of MspA in the bilayer. Due to the presence of polar amino-acid side-chains at the exterior of the MspA’s “head”, hydrogen bonding [29] is most likely responsible for the discrepancy of the calculated packing parameter P = 0.31 and the experimental finding that vesicles are formed, which requires 0.5 < P < 1. Charge attraction/repulsion [30] apparently only plays a minor role, since the observed formation of liposomes does not strongly depend on the ionic strengths of the aqueous medium. The anisotropy of the negative and positive charges at the outer surface of MspA is shown in Figure 1B and Figure 1C. The experimental finding that MspA forms vesicles and not micelles under the described conditions clearly indicates that there exist additional forces in supramolecular MspA aggregates, which are hydrogen bonding and, to a significantly lesser extent, charge attraction. Thus, the transfer of MspA from the aqueous phase to the bilayer is driven by the hydrophobic effect, which is the thermodynamic driving force of vesicle formation. The influence of charge attraction/repulsion and hydrogen bonding will be discussed below.

### Zeta potentials of MspA-vesicles as functions of temperature and ionic strength

**Figure 7:**
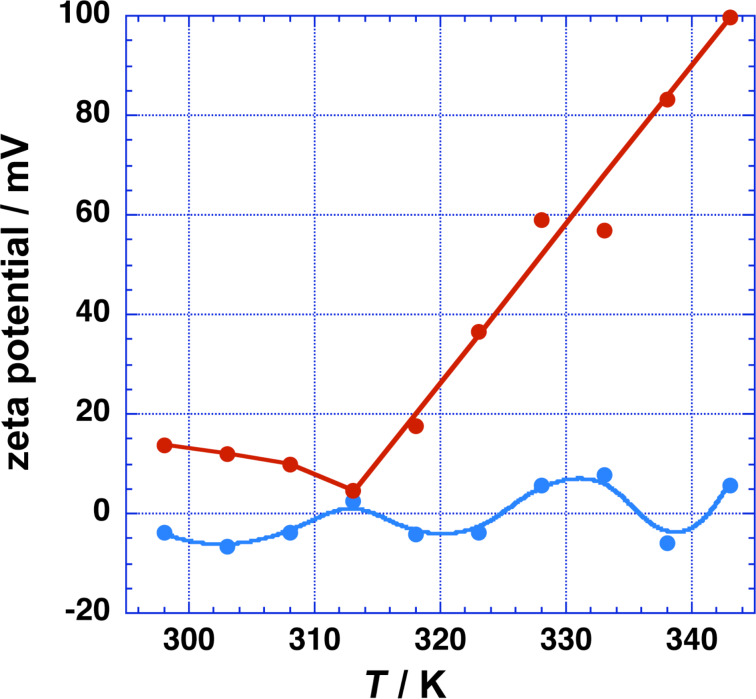
Zeta potential of MspA aggregates as a function of temperature. Blue: MspA (1.688 × 10^−5^ mg·mL^−1^) in (5 × 10^−5^)× PBS; red: MspA (1.688 × 10^−5^ mg·mL^−1^) in 1× PBS.

To study the charge of the MspA vesicles as a function of temperature, we performed a series of zeta-potential measurements by electrophoretic light scattering [31]. The zeta potential is the electric potential between the slipping plane in the interfacial double layer and the bulk solution [31]. The results of the measurements are summarized in Figure 7.

The zeta potential of MspA vesicles oscillates around the point of zero charge in (5 × 10^−5^)× PBS as the temperature increases. The observed oscillations are reproducible (experimental error: ±5 mV at each respective temperature). They are indicative of a complicated interplay between deprotonation of the carboxylic acid groups of MspA and increased protonation of its amine functions. Both effects increase with increasing temperature. The enhanced macromolecular motion of MspA with increasing temperature may lead to a changing dynamics of the forming and breaking of hydrogen bonds as the temperature is increased. We are unable at this point to provide a qualitative analysis of this phenomenon.

The zeta potential of MspA vesicles in 1× PBS as a function of temperature is completely different. ζ is slightly positive (ζ = 10 ± 14 mV) in the temperature range from 296 to 320 K. Beyond 320 K, a remarkable increase in ζ is observed. At 344 K, ζ = 100 ± 12 mV indicates an excellent stabilization of the MspA vesicles in PBS. The temperature dependence of ζ is indicative of an endergonic adsorption process of cations (Na^+^ and K^+^) at MspA. The observed increase in ζ as a function of *T* is completely reversible. It is noteworthy that the remarkable difference in the surface charges of MspA vesicles in diluted PBS and 1× PBS only results in slightly different diameters, as shown in Figure 2. The size of the MspA vesicles decreases in both media; however, the decrease is stronger in diluted PBS than in 1× PBS, indicating that charge attraction/repulsion does not contribute significantly to (Δµ^0^/*k*_B_*T*)_head groups_, although it is the strongest interactive force (±5–8 kJ·mol^−1^ per bridge/repulsion) in supramolecular binding [30]. The pH of both media ((5 × 10^−5^)× PBS and 1× PBS) was exactly 7.20 at 296 K. Therefore, we assume that the extent of hydrogen bonding events between the MspA “heads” in the bilayer occurs when forming vesicles from both media. Hydrogen bonds between side chains of proteins have a typical strength of 4–5 kJ·mol^−1^ per bridge [23]. At this point we cannot distinguish between the effects of charge attraction/repulsion and hydrogen bonding on the supramolecular attraction of the vestibules of MspA when forming the bilayer. In addition, different types of attraction/repulsion may exist between MspA molecules on the same and the opposite side of the bilayer, because the charge distribution at the surface of MspA is not isotropic (Figure 1). The increase of the diameter of the vesicles in both diluted and standard PBS between 296 K and 312 K (1× PBS) or 318 K ((5 × 10^−5^)× PBS) could be caused by a thermal activation step required for vesicle formation. Due to the thermal stability of MspA, it is reasonable to assume that the number of vesicles decreases while their diameters increase, because the concentration of free MspA will be very low. Since MspA is a large surfactant, the requirement for thermal activation is comprehensible. It should also be noted that many classic vesicles/liposomes are not in their thermodynamic minimum [32].

## Conclusion

TEM has provided experimental evidence that the mycobacterial porin MspA forms vesicles at low concentrations from aqueous buffers. The size of the MspA vesicles is strongly dependent on temperature, but not on the salt content of the aqueous buffer. The hydrodynamic maximum of the vesicles has been determined by dynamic light scattering to be approximately 180 nm. It occurs at 312 K (standard PBS) and 318 K (diluted PBS). The occurrence of a temperature maximum is indicative of a thermal activation step required for the formation of bilayers from MspA, which is a rather large surfactant of 9.6 nm in length and 8.8 nm in diameter. Increasing the temperature favors reversible cation (Na^+^, K^+^) adsorption at MspA in 1× PBS. It is noteworthy that the corresponding significant increase in ζ does not significantly affect the hydrodynamic diameter of the vesicles. The aggregation number of the vesicles formed by MspA varied between *N* = 1395 and *N* = 2470 for the diameters measured by DLS. Although the aggregation behavior of MspA as a function of temperature is apparently governed by the hydrophobic effect, we have observed evidence for a strong influence of the ionic strength in the surface charges of MspA vesicles. Our experimental data clearly indicate that temperature is an important experimental variable in this supramolecular system formed by a stable protein. Advances in protein design will lead to increasingly stable supramolecular systems using proteins as biological building elements in functional nanoscopic systems. It is our prediction that the physical properties of these systems will be strongly dependent on their temperature. This is of equal importance for their assembly as well as for their function under operating conditions.

## Experimental

MspA was extracted from *M. smegmatis* and purified, adapting a procedure that was originally developed by Niederweis and co-workers [21–22]. The procedure is described in detail in Supporting Information File 1. The hydrodynamic diameter and the zeta potential of the MspA aggregates were measured on a ZetaPALS Zeta Potential Analyzer (Brookhaven Instruments Corporation) by hydrodynamic light scattering and laser Doppler electrophoresis. One drop (50 μL) of wild type MspA extract (0.224 mg/mL in 1× PBS) was diluted in 2.0 mL deionized water and the average effective diameter of protein aggregates was recorded while increasing the temperature of the sample. The measurements were taken at increasing temperature values from 25 to 70 °C at intervals of 5 °C. A consistent fluctuation of the effective diameter was observed with increasing temperature. The experiment was repeated using 2.0 mL of 1× PBS buffer solution instead of deionized water. Similarly, the zeta potential was measured for wild type MspA extracts in both deionized water and 1× PBS solutions. Transmission electron micrographs were recorded in the Microscopy and Analytical Imaging Laboratory of the University of Kansas, 1043 Haworth Hall, 1200 Sunnyside Ave, Lawrence, KS 66045. The morphology of MspA aggregates from aqueous buffers was characterized by transmission electron microscopy (TEM). The TEM were prepared by immersing carbon-coated 200-mesh copper grids in aqueous liposome-containing solutions, followed by counter-staining by 2% aqueous uranyl acetate solution, and overnight drying in a desiccator. The dried grids were analyzed by using a HRTEM FEI Tecnai F20 XT Field Emission Transmission Electron Microscope 200 kV, operated at 80 kV.

## Supporting Information

The Supporting Information section contains the procedure for extraction and purification of MspA, the general formula for calculating the zeta potentials and representative results from dynamic light scattering.

File 1 Detailed experimental data.
