# Supplementary material for: Nanoscopic surfactant behavior of the porin MspA in aqueous media
Source: Beilstein J Nanotechnol. 2013 Apr 25;4:278–84. doi: 10.3762/bjnano.4.30 (PMC3678404; doi:10.3762/bjnano.4.30)
Supplement: File 1 — Detailed experimental data. [file Beilstein_J_Nanotechnol-04-278-s001.pdf]

Supporting Information

for

Nanoscopic surfactant behavior of the porin MspA in aqueous media

Ayomi S. Perera*[1], Hongwang Wang[1], Tej. B. Shrestha[2], Deryl L. Troyer[2] and Stefan H. Bossmann*[1]

Address: [1]Kansas State University, Department of Chemistry, CBC Building 201, Manhattan, KS 66506, USA and [2]Kansas State University, Department of Anatomy & Physiology, Coles 130, Manhattan, KS 66506, USA

Email: Stefan H. Bossmann* - sbossman@ksu.edu; Ayomi S. Perera* - ayomee@ksu.edu

* Corresponding author

Detailed experimental data

1. Extraction and purification of MspA

M. smegmatis cultures (mc^2 155, a gift of Dr. Michael Niederweis at the University of Alabama, Birmingham) were inoculated onto Agar plates by the streaking method and kept in an incubator at 37.5 °C for 3–4 days until colonies developed. Liquid media 7H9 broth base was prepared for inoculation of the colonies. Into a 1000 mL beaker, 4.2 g of Middlebrook 7H9 broth base and

3 mL of 60% glycerol, followed by 900 mL of distilled water were filled. The solution was stirred into a homogeneous mixture, poured into 1 L Nalgene bottles and autoclaved, then stored at 4 °C. For inoculation of small bacterial cultures, 50 mL of the 7H9 broth was mixed with 125 µL of 20% Tween 80 and 50 µL of hygromycin (prepared to a 50 µg/µL aqueous solution) under a laminar flow and vortexed for 1 min. Two 10 mL culture tubes were each filled with 5 mL of this medium and the *M. smegmatis* colonies were transferred via a sterilized needle into each tube. These were kept in a shaker at 37.5 °C and 75 rpm for 48 h to obtain mature cultures. After 48 h, 0.5 mL of growth, the cultures from each tube were transferred into two new culture tubes and mixed with 4.5 mL of prepared 7H9 media in order to refresh the cultures.

Scale up of the production of *M. smegmatis* cultures was achieved by inoculating the 'small' cultures prepared above into 'medium' and 'large' cultures. The 'medium' cultures were prepared by transferring 2 mL each from 'small' culture media (48 h old) into a 125 mL culture flask adding up to 4 mL of culture-media. 36 mL of prepared growth media were added to this flask (total volume of 40 mL) and kept in shaker for 48 h. 'Large' cultures were prepared by transferring 40 mL of 'medium' culture into a 2 L culture flask followed by addition of 2.25 mL of 20% Tween 80 and 300 µL of hygromycin. Next 1 L of 7H9 broth was added to this flask and kept in shaker for 48 h. All media preparations and inoculations were performed under sterile conditions under laminar flow.

1.1 Extraction and HPLC purification of MspA from *M. smegmatis*

Two 48 h old 'large' culture flasks were emptied into large centrifuge bottles and centrifuged at 3696 rpm in 5 °C for 1 h to separate the cells from the medium. The supernatant was discarded and 50 mL of PBS buffer was added to the cells and vortexed until dispersed. The mixture was then centrifuged at 10016 rpm at 5 °C for 1 h then the supernatant was discarded and the cells were weighed. 3.5 mL of PEN buffer was added for every 1 g of cells. *n*-Octyl-oligo-oxyethylene (*n*OPOE) detergent was added as 0.08% v/v of nOPOE/PEN mixture to obtain the maximum extraction of proteins. The mixture was then heated at 65 °C for 1 h under stirring. Afterwards, the mixture was cooled for 5 min in a freezer and centrifuged at 10016 rpm for 1 h. The supernatant obtained was separated out and mixed with an equal volume of ice-cold acetone and kept in the freezer (0 °C) overnight in order to precipitate the protein. The next day the mixture was centrifuged for 30 min at 10016 rpm at 5 °C. The supernatant was discarded and the precipitate was dissolved in PBS. The solution was then poured into 3000 MWCO ultra-filtration tubes and concentrated by centrifuging at 10016 rpm for 30–60 min until a constant volume was obtained. The protein extract was then stored at 4 °C followed by analysis via gel electrophoresis.

HPLC was performed using a POROS® column of 10 × 10 mm (Purchased from Applied Biosystems) and detection was carried out at 254 nm with a flow rate of 0.5 mL/min. Buffer A (for H_2O 1000 mL, HEPES 25 mM, NaCl 10 mM, 0.5% OPOE 500 µL, pH 7.5) and buffer B (for H_2O 1000 mL, HEPES 25 mM, NaCl 2 mM, 0.5% OPOE 500 µL, pH 7.5) were used as solvents. Concentrations of wild type extracts were determined by the Bradford assay [1].

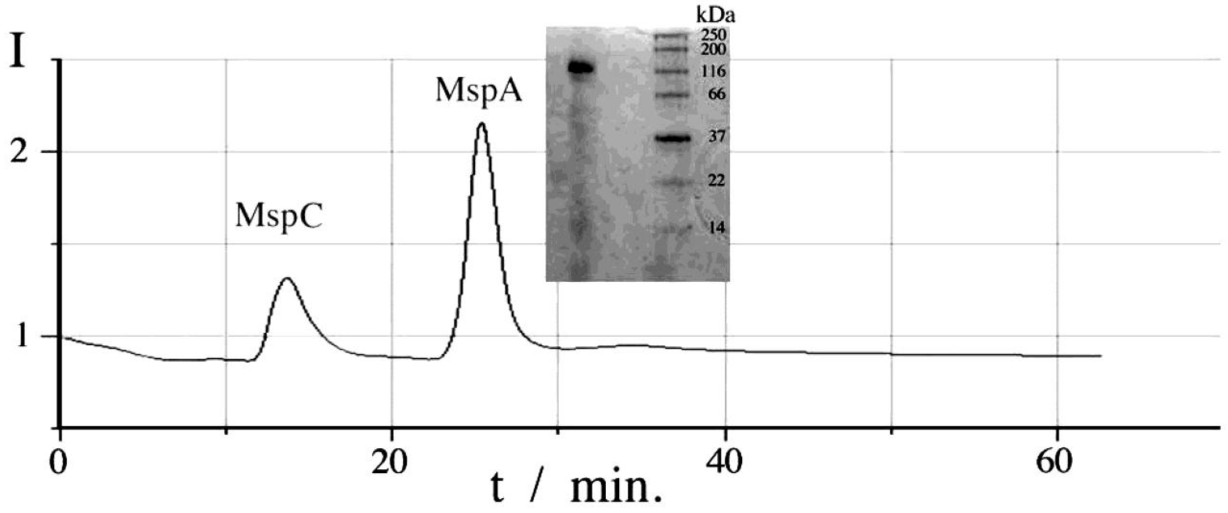

Figure S1: HPLC purification of MspA, extracted from *M. smegmatis* (mc^2 155), I: arbitrary signal intensity (refractive index detection).

Two peaks were observed from HPLC. Peak 1 was identified as MspC, based on literature [2]. Peak 2 was identified as MspA. MspA was dialyzed for 24h against water and for another 24h against PBS using Snake Skin Tubing (Thermo Scientific), molecular weight cut-off: 7 k. HPLC- and mass-spectroscopy-analysis of the final MspA did not yield any indication of the presence of surfactant molecules in the aqueous solutions of MspA.

2. Typical polydispersities of the MspA vesicles

WTMspA_H2O_25 (Combined)
Mar 22, 2012 14:14:12

Effective Diameter: 137.9 nm
Polydispersity: 0.078
Avg. Count Rate: 332.9 kcps
Baseline Index: 8.4/100.00%
Elapsed Time: 00:00:45

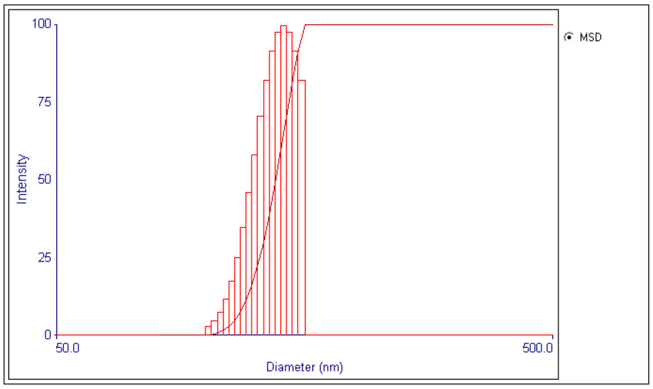

Run	Eff. Diam. (nm)	Half Width (nm)	Polydispersity	Baseline Index
1	134.6	50.2	0.139	8.6/100.00%
2	137.8	7.6	0.003	9.0/100.00%
3	140.6	46.0	0.107	5.4/100.00%
Mean	137.7	34.6	0.083	7.6/100.00%
Std. Error	1.7	13.5	0.041	1.1/ 0.00
Combined	137.9	38.6	0.078	8.4/100.00%

Figure S2: Dynamic light scattering (DLS) of MspA vesicles (MspA: 1.688×10^{-5} mg·mL^{-1} in $(5 \times 10^{-5})\times$ PBS) at 298 K.

WTMspA50-40 (Combined)
Mar 16, 2012 13:08:37

Effective Diameter: 150.4 nm
Polydispersity: 0.050
Avg. Count Rate: 217.5 kcps
Baseline Index: 2.0/100.00%
Elapsed Time: 00:00:45

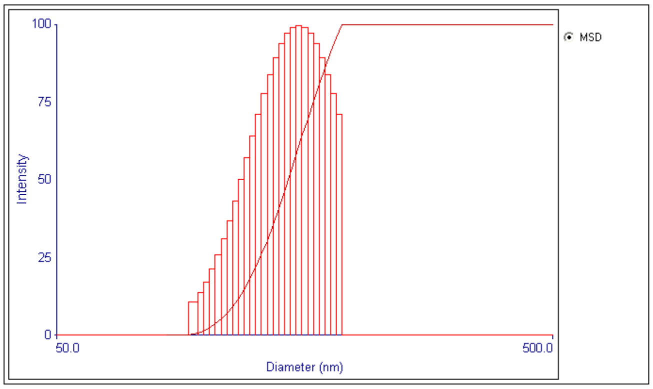

Run	Eff. Diam. (nm)	Half Width (nm)	Polydispersity	Baseline Index
1	150.1	47.6	0.101	8.5/100.00%
2	152.2	48.7	0.102	0.0/100.00%
3	147.0	10.4	0.005	3.4/100.00%
Mean	149.8	35.6	0.069	4.0/100.00%
Std. Error	1.5	12.6	0.032	2.5/ 0.00
Combined	150.4	33.5	0.050	2.0/100.00%

Figure S3: Dynamic light scattering (DLS) of MspA vesicles (MspA: 1.688×10^{-5} mg·mL^{-1} in $(5 \times 10^{-5})\times$ PBS) at 313 K.

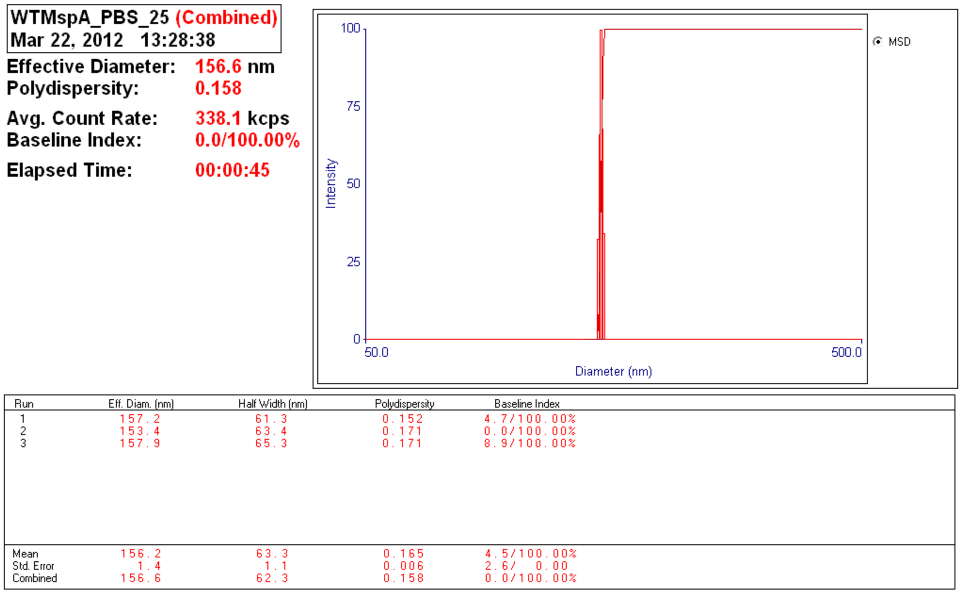

Run	Eff. Diam. (nm)	Half Width (nm)	Polydispersity	Baseline Index
1	157.2	61.3	0.152	4.7/100.00%
2	153.4	63.4	0.171	0.0/100.00%
3	157.9	65.3	0.171	8.9/100.00%
Mean	156.2	63.3	0.165	4.5/100.00%
Std. Error	1.4	1.1	0.006	2.6/ 0.00
Combined	156.6	62.3	0.158	0.0/100.00%

Figure S4: Dynamic light scattering (DLS) of MspA vesicles (MspA: 1.688 × 10^{-5} mg·mL^{-1} in 1× PBS) at 298K.

3. Polydispersities of the MspA vesicles versus temperature and size

The polydispersities, as determined by DLS, are plotted versus the temperature in Figure S5.

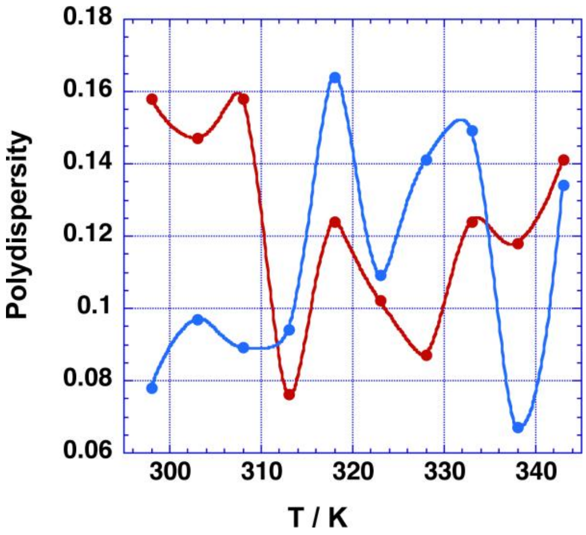

Figure S5: Polydispersities of MspA aggregates as a function of temperature: blue: MspA (1.688 × 10^{-5} mg·mL^{-1} in (5 × 10^{-5})× PBS; red: MspA (1.688 × 10^{-5} mg·mL^{-1} in 1× PBS).

The dependencies of the polydispersity on the temperature and on the vesicle diameter have been experimentally reproduced. The relative experimental error in diameter has been determined to be ±8 nm; the relative error in polydispersity is ±0.03. The data shown in Figure S5 is indicative of a highly dynamic supramolecular behavior, which is highly dependent on temperature.

4. Size of MspA vesicles in dependence of MspA concentration

In 1× PBS there is an increase in particle size at 1.688×10^{-5} mg·mL^{-1} of MspA; however, with further increase in concentrations the particle size decreases at first and then remains constant. The overall particle-size change is only in the narrow range of 107–127 nm. In diluted PBS there is a gradual increase of particle size with increase in MspA concentration in the range from 98 to 116 nm. Both media show similar particle size at the highest concentration. It is reasonable to state that the overall particle size of MspA vesicles are not subject to significant changes in particle size with increasing concentration or in different media. This is a marked difference from the results of the temperature-dependent study.

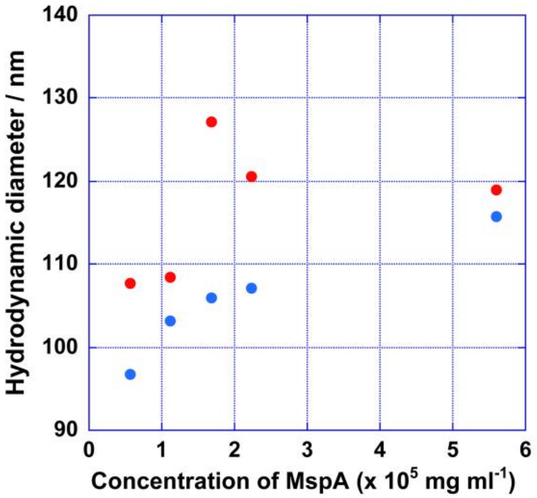

Figure S6: Hydrodynamic diameters of MspA aggregates as a function of concentration ($\times 10^5$ mg·mL^{-1}): blue: MspA in $(5 \times 10^{-5})\times$ PBS; red: MspA in 1× PBS.

5. Measurement of the zeta potentials

Under controlled experimental conditions, the zeta potential, ζ, is proportional to the electrophoretic mobility. The zeta potential (expressed in mV) is the parameter, which determines the strength of the attraction versus repulsion between nanoparticles. The relationship between mobility and zeta potential is a function of the following parameters:

$$\mu_{ep} = \varepsilon_r \varepsilon_0 \frac{\zeta}{\eta} f(ka) \tag{1}$$

m_{ep} = electrophoretic mobility (micron/second)/(volt/cm),
e_r = relative permittivity of the liquid (here: PBS)
e_0 = relative permittivity of the vacuum
h = viscosity of the liquid
The factor *f*(*ka*) is of the order unity.

References

1. Kruger, N. J., The protein protocols handbook, 2nd ed.; Walker, J. M., Ed.; Humana Press Inc.: Totowa, NJ, 2002.
2. Heinz, C.; Niederweis, M. *Anal. Biochem*. **2000,** *285,* 113–120.
